# The Potential of Natural Carotenoids-Containing Sericin of the Domestic Silkworm *Bombyx mori*

**DOI:** 10.3390/ijms25073688

**Published:** 2024-03-26

**Authors:** Sirui Liu, Qing Zhang, Hanyue Zhou, Bo Zhang, Ming Yu, Yonglong Wang, Yanqun Liu, Chunli Chai

**Affiliations:** 1 College of Sericulture, Textile and Biomass Sciences, Southwest University, Chongqing 400715, China; lsr970801@email.swu.edu.cn (S.L.); zhq20190810@email.swu.edu.cn (Q.Z.); naruto123@email.swu.edu.cn (H.Z.); zb2333@email.swu.edu.cn (B.Z.); senkrecht@email.swu.edu.cn (M.Y.); suw959756564@email.swu.edu.cn (Y.W.); 2 College of Bioscience and Biotechnology, Shenyang Agricultural University, Shenyang 111000, China; liuyanqun@syau.edu.cn; 3 State Key Laboratory of Resource Insects, Key Laboratory of Sericultural Biology and Genetic Breeding, Ministry of Agriculture and Rural Affairs, College of Sericulture, Textile and Biomass Sciences, Southwest University, Chongqing 400715, China

**Keywords:** sericin, carotenoids, physicochemical properties

## Abstract

Sericin derived from the white cocoon of *Bombyx mori* has been attracting more attention for its utilization in food, cosmetics, and biomedicine. The potential health benefits of natural carotenoids for humans have also been well-established. Some rare strains of *Bombyx mori* (*B. mori*) produce yellow–red cocoons, which endow a potential of natural carotenoid-containing sericin. We hypothesized that natural carotenoid-containing sericin from yellow–red cocoons would exhibit better properties compared with white cocoon sericin. To investigate the physicochemical attributes of natural carotenoid-containing sericin, we bred two silkworm strains from one common ancestor, namely XS7 and XS8, which exhibited different cocoon colors as a result of the inconsistent distribution of lutein and β-carotene. Compared with white cocoon sericin, the interaction between carotenoids and sericin molecules in carotenoid-containing sericin resulted in a unique fluorescence emission at 530, 564 nm. The incorporation of carotenoids enhanced the antibacterial effect, anti-cancer ability, cytocompatibility, and antioxidant of sericin, suggesting potential wide-ranging applications of natural carotenoid-containing sericin as a biomass material. We also found differences in fluorescence characteristics, antimicrobial effects, anti-cancer ability, and antioxidants between XS7 and XS8 sericin. Our work for the first time suggested a better application potential of natural carotenoid-containing sericin as a biomass material than frequently used white cocoon sericin.

## 1. Introduction

*Bombyx mori* silk is one of the valuable biological materials due to its natural abundance, high mechanical strength, and excellent biocompatibility [1]. It is widely used in biomedical materials, cosmetics, and food [2]. Structurally, silk comprises the inner core of fibroin (70–80%) and the outer layer of sericin (20–30%) along with other substances such as waxes and pigments [3]. Due to the favorable antioxidant properties, antibacterial properties, UV protection, excellent water solubility, and moisture retention [4], sericin has been used to prepare silk sericin-based biomaterials (SS-based biomaterials) by cross-linking, copolymerization, and combining with other polymers [5]. The forms of silk sericin-based biomaterials include nanoparticles, hydrogel, scaffolds, films, and microneedles [6]. At present, the most popular research and application of SS-based biomaterials are focusing on vitro/vivo monitoring, drug delivery, or wound healing [7]. Membranes, hydrogels, or scaffolds made from SS provide necessary mechanical support and physical protection for tissue injuries. Meanwhile, SS-based particles can deliver and release therapeutic agents on demand [6,8].

Carotenoids play crucial roles in animals and plants as precursors of vitamin A, antioxidants, and colorants [9]. They are often involved in physiological activities such as cell differentiation, metabolic regulation, reproductive development, and immune regulation [10]. Carotenoids usually contain eight isoprenoid units as their basic components, which endow carotenoids with good properties [11]. The potential health benefits of carotenoids have been well-established in many studies, for example anticancer, anti-inflammatory, antibacterial, antidiabetic, and neuroprotective [12]. Based on the physiological activity of carotenoids, they are embedded in drug carriers for medical research [13].

Our question is whether the natural incorporation of carotenoids into sericin will improve its unique structural characteristics and properties. Colored cocoon silk is rich in natural pigments such as lutein, β-carotene, and flavonoids [14,15]. The accumulation of carotenoids in silk sericin contributes to the pigmentation of yellow–red cocoons as yellow, red, pink, or straw [15]. Differences in the type and content of carotenoids in silkworm silk glands are responsible for the coloration of cocoons. In previous research, the raw material for sericin used was derived from white cocoons, while no reports on the use of sericin from naturally colored cocoon resources were noted [4,16].

The aim of this study was to compare the physicochemical, antimicrobial properties, and biocompatibility of natural carotenoid-containing sericin with frequently used white cocoon sericin. For this purpose, two silkworm strains spinning yellow–red cocoons were selectively bred for over 12 generations from one common ancestor, named XS7 and XS8. The two strains and a white cocoon strain Qiubai were used to obtain the natural carotenoid-containing sericin and white cocoon sericin, respectively. The antibacterial effect, anti-cancer ability, cytocompatibility, and antioxidant of sericin were comparatively investigated between natural carotenoid-containing sericin and white cocoon sericin, and between XS7 and XS8. Our work confirmed the potential of new natural carotenoid-carrying sericin as biomass material and provided evidence of the differences in physicochemical properties between different carotenoid-carrying strains.

## 2. Results

### 2.1. Breeding of Silkworm Strains XS7 and XS8

**Figure 1:**
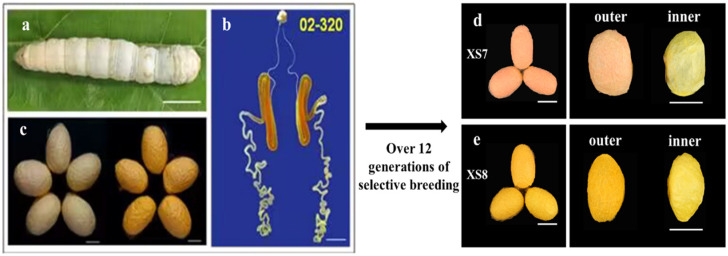
Breeding selection of strains XS7 and XS8 from 02-320. (**a**) Larvae of 02-320; (**b**) silk glands of 02-320; (**c**) cocoons of 02-320; (**d**) cocoons of XS7; and (**e**) cocoons of XS8. Scale bar: 1 cm.

We selectively bred two silkworm strains, XS7 and XS8, from one common ancestor strain 02-320, based on cocoon color, for more than 12 generations (Figure 1). Strain 02-320 is one of the silkworm strains conserved in the National Silkworm Genetic Resource GenBank, Southwest University, China. Unlike four-molter silkworms widely used in production, 02-320 belongs to the three-molter silkworm. The larval body color of 02-320 is white and the middle silk glands exhibit a bright yellow–red color. However, it spins cocoons with inconsistent colors, some are yellow and others light red. Typical red and yellow cocoons were harvested separately, and self-fertilization was used for the breeding of the next generation. Eventually, we obtained two silkworm strains, XS7 and XS8, with red and yellow cocoons, respectively. Further analysis revealed that for strain XS7 the outer and inner cocoon is red and golden yellow, while for strain XS8 the outer and inner cocoon is dark yellow and golden yellow (Figure 1).

### 2.2. Differences in Cocoon Color between XS7 and XS8

**Figure 2:**
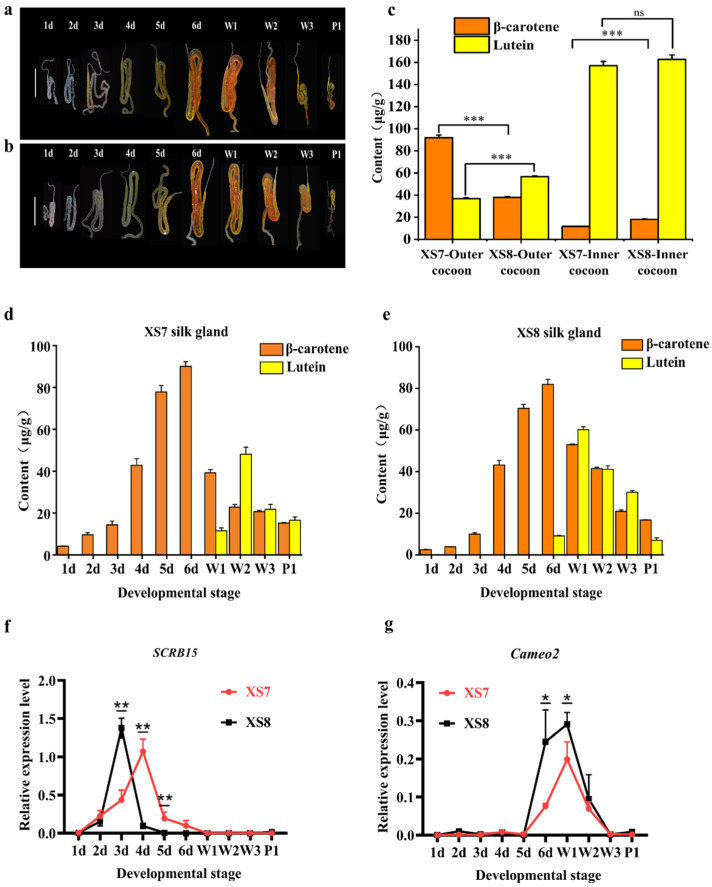
Color differences between XS7 and XS8 cocoons. (**a**) XS7 silk glands; (**b**) XS8 silk glands; (**c**) carotenoid content in outer and inner layers of cocoons; (**d**) carotenoid content in XS7 silk glands; (**e**) carotenoid content in XS8 silk glands; (**f**) relative expression level of *SCRB15*; and (**g**) relative expression level of *Cameo2*. 1 d–6 d, 1st–6th day of fourth instar; W1-W3, 1st–3rd day of wandering; P1, 1st day of pupation. Scale bar: 1 cm. Each bar represents the mean ± SD of three samples. *, *p* < 0.05; **, *p* < 0.01; ***, *p* < 0.001; ns, no significance.

To explore the reasons for differences in cocoon color between XS7 and XS8, we first dissected silk glands and examined the changes in size and color during the larval stage of the final (fourth) instar, as shown in Figure 2a,b. During the initial two days, the silk glands presented a milky white appearance, with no discernible pigmentation in either strain. Starting from the 3rd day, the middle silk glands exhibited remarkable changes in both size and color in accordance with the development of the larva. Silk glands were enlarged and their colors were intensified. The middle silk gland had the biggest size on the 6th day. The color was changed from light yellow to golden yellow and red, indicating a gradual accumulation of pigments within the middle silk gland. Silk glands were atrophied until invisible due to cocooning and apoptosis of silk gland cells during the metamorphosis from larva to pupa. Meanwhile, the silk glands still appeared light yellow, suggesting that some pigments were still reserved in the silk gland cells until the silk glands were completely degraded. Overall, the alterations of silk glands throughout the larval final instar did not show significant differences between strains XS7 and XS8.

Secondly, the content and type of carotenoids in the silk glands of XS7 and XS8 were analyzed using RP-HPLC-DAD. In XS7, the predominant carotenoid in the silk glands of the final instar was β-carotene (Figure 2d). From the initial day of the wandering stage, lutein gradually accumulated in the silk gland of XS7 (Figure 2d). In XS8, β-carotene served as the chief carotenoid in the silk glands from the first to the fifth day of the final instar. A gradual increase in lutein levels in the silk glands of XS8 started from the 6th day of the final instar. From the 2nd day of the wandering stage to 1st day of pupation, the contents of β-carotene and lutein decreased gradually in the silk glands of both strains (Figure 2e).

The cocoon layer color was consistent with the composition and content of pigments, as shown in Figure 2c. In the outer layer of the XS7 cocoon, the content ratio of β-carotene to lutein was about 2.6:1, so the outer cocoon layer of XS7 appeared light red (Figure 1d). Within the inner layer of the XS7 cocoon, the content ratio of β-carotene to lutein was drastically shifted to approximately 1:13.4. The significant surge in lutein content compared to β-carotene resulted in a golden yellow inner cocoon layer in XS7 (Figure 1d). In the outer cocoon layer of XS8, the content ratio of β-carotene to lutein was about 1:1.5, distinct from the carotenoid composition and content in the outer cocoon layer pigment of XS7. The content of β-carotene in the outer cocoon layer of XS8 was significantly lower than that of XS7 (*p* < 0.001). Moreover, the content of lutein was significantly higher than that of XS7 (*p* < 0.001). Thus, the outer cocoon layer of XS8 appeared dark yellow (Figure 1e). In the inner cocoon layer of XS8, the content ratio of β-carotene to lutein was about 1:9, which was similar to the composition and content of carotenoids in the inner cocoon layer pigment of XS7 cocoon. Therefore, the inner layer of the cocoon also appeared gold–yellow (Figure 1e).

Several key proteins that play crucial roles in the selective transportation of carotenoids to silk glands have been identified in yellow–red cocoon strains [17]. Early studies revealed that Cameo2 was responsible for transporting lutein into middle silk glands, while SCRB15 specially bonded and transported β-carotene [17]. To further clarify the forming mechanism of the coloration difference between the inner and outer cocoons of XS7 and XS8, the expression profile of *SCRB15* and *Cameo2* was detected by qRT-PCR. We found that *SCRB15* was expressed in the silk gland of XS7 during the 2nd–6th day of the final instar, and the highest expression was observed on the 4th day of the final instar (Figure 2f). *Cameo2* was expressed in the silk glands of XS7 from the 6th day of the final instar to the 2nd day of the wandering stage, and the highest expression was observed on the 1st day of the wandering stage (Figure 2g). Contrasting with XS7, *SCRB15* was expressed in the silk gland of XS8 during the 2nd–4th day of the final instar, and the highest expression was observed on the 3rd day of the final instar in XS8 (Figure 2f). *Cameo2* expression was detected in the silk glands of XS8 from the 6th day of the final instar to the 2nd day of the wandering stage, reaching a maximum on the 1st day of the wandering stage. Obviously, there was a higher expression of *Cameo2* on the 6th day of final instar in XS8 than that in XS7 (Figure 2g). The gene expression analyses confirmed that XS7 and XS8 were two distinct silkworm strains, with significant differences in color. We thus used XS7 and XS8 sericin to further explore the differences between white cocoon sericin and yellow–red cocoon sericin.

### 2.3. Comparison of Sericin from XS7, XS8 and Qiubai

**Figure 3:**
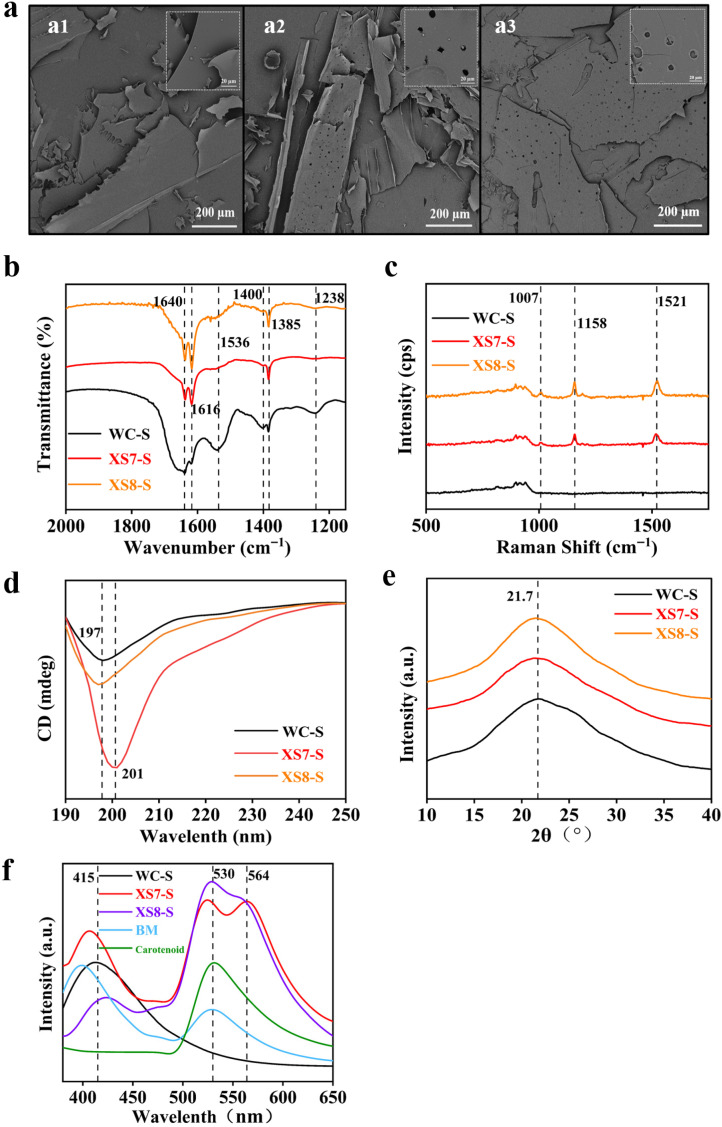
Characterization of physicochemical properties of sericin. (**a1**) SEM image of Qiubai sericin; (**a2**) SEM image of XS7 sericin; (**a3**) SEM image of XS8 sericin; (**b**) FT-IR spectrum; (**c**) Raman spectrum; (**d**) CD spectrum; (**e**) XRD spectrum; and (**f**) fluorescence spectrum. WC-S, white cocoon sericin; XS7-S, XS7 sericin; XS8-S, XS8 sericin; BM, sericin-carotenoids blending material; carotenoids, lutein, and β-carotene standards.

The external morphology of freeze-dried sericin from XS7, XS8, and Qiubai was observed by the scanning electron microscope (Figure 3a). Sericin from XS7 and XS8 (yellow–red cocoon) presented a similar lamellar and loose structure to Qiubai (white cocoon). All of them were lightweight and easy to break and collapse. However, sericin from XS7 and XS8 showed more pores compared with sericin from Qiubai.

The structural difference of sericin from XS7, XS8, and Qiubai was investigated by the FT-IR spectra (Figure 3b). Sericin extracted from XS7, XS8 and Qiubai all showed absorption peaks at 1238 cm^−1^, 1385 cm^−1^, 1400 cm^−1^, 1536 cm^−1^, 1616 cm^−1^, 1640 cm^−1^. The absorption peaks at 1238 cm^−1^ and 1400 cm^−1^ belong to the C-N stretching of the amide III band [18,19]. The absorption peaks at 1385 cm^−1^ and 1536 cm^−1^ originated mainly from the C-H stretching vibration and in-plane N-H bending of the amide II band [20]. The overlapping peaks at 1616 cm^−1^ and 1640 cm^−1^ were due to C=C, C=O stretching vibration of the amide Ι band, corresponding to a β-sheet conformation [20,21]. Sericin from XS7, XS8, and Qiubai possessed the same intensity peak at 1385 cm^−1^ (CH_3_) [19]. However, the intensity of the absorption bands of amide I in XS7, and XS8 sericin (1616 cm^−1^) was increased, while that of amide I (1640 cm^−1^) was decreased than that in Qiubai sericin. The results suggested that the secondary structure of XS7 and XS8 sericin tended to shift from a random coil to a β-sheet [20,22]. In addition, compared with Qiubai sericin, the peak intensity at 1536 cm^−1^ (N-H bending) in amide II, at 1238 cm^−1^ (C-N stretching) in amide III, 1400 cm^−1^ (C-N stretching) showed a significant decrease in both XS7 and XS8 sericin group. These results suggested that the chemical bonds in sericin have changed or been disrupted by the incorporation of carotenoids. We further analyzed the secondary structure of different sericin samples by the software Peakfit v4.12 (Cranes Software, San Jose, CA, USA). Results showed that β-sheet increased significantly in sericin proteins of XS7 and XS8 compared to white cocoon sericin (Table 1). The results indicated that the molecular conformation of sericin has changed due to the incorporation of carotenoids. Taken together, we found obvious differences in the molecular structure between sericin from white cocoon (Qiubai) and carotenoid-containing sericin from yellow–red cocoon (XS7, XS8), but no significant differences between XS7 and XS8 sericin.

The chemical structure of sericin from XS7, XS8, and Qiubai was analyzed by the Raman spectrum (Figure 3c). Compared with white cocoon (Qiubai) sericin, the Raman spectrum of XS7 and XS8 sericin showed three distinct characteristic peaks of the polyolefin structure of carotenoids at 1007 cm^−1^, 1158 cm^−1^, and 1521 cm^−1^. The characteristic peak of 1007 cm^−1^ was caused by a swing in methyl [23]. The characteristic peak of 1158 cm^−1^ belonged to the stretching vibration of the C-C [24]. In addition, the characteristic peak of 1521 cm^−1^ was due to the stretching vibration of the C=C in the polyene chain [23]. The results revealed that the carotenoid-containing sericin extracted from XS7 and XS8 had similar protein structures.

The interaction between carotenoids and sericin, and the differences in the secondary structures of sericin from XS7, XS8, and Qiubai were detected by the circular dichroism (CD) analysis (Figure 3d). We observed a negative band at 197 nm in sericin from Qiubai and XS8, but a strong signal negative band at 201 nm in sericin from XS7. The results showed that all the secondary structures of XS8, XS7, and Qiubai sericin contained a large amount of random coil structure [20,25]. The results also indicated that XS7 may possess a unique disordered coiled secondary structure, distinct from XS8 and Qiubai sericin.

The crystal structure of sericin extracted from XS7, XS8, and Qiubai was characterized by an X-ray diffraction (Figure 3e). All the X-ray powder diffraction curves of the three samples showed a broad hump at about 21.7°, which meant that the three kinds of sericin were amorphous [26]. The result indicated that carotenoids did not influence the crystalline structure of sericin.

Fluorescence spectra of sericin from XS7, XS8, and Qiubai were obtained by a fluorescent spectrometer excited at 340 nm. Samples of lutein and β-carotene standards and blending of white cocoon sericin with carotenoid were used as control. We found an emission peak at 530 nm in the fluorescence spectrum of carotenoid, 415 nm in the spectrum of white cocoon sericin, and 415 nm and 530 nm in the spectrum of blending of white cocoon sericin and carotenoid. Notably, we observed a new emission peak at 564 nm in the spectrum of XS7 and XS8 sericin, rather than in sericin–carotenoids blend material (Figure 3f). XS8 sericin had a stronger absorption peak at 530 nm and a weaker absorption peak at 564 nm compared with XS7 sericin. These results suggested that the emission peak at 564 nm may be due to the interaction of sericin with carotenoids, and the carotenoid-containing sericin from XS7 and XS8 is not a simple blend of pure sericin and carotenoid. Sericin and carotenoids may be incorporated together by chemical bands in the silk glands of silkworm larvae or during the process of spinning. The differences in carotenoid composition may influence the fluorescence characteristics of sericin.

### 2.4. Comparison of Antimicrobial Effects of Sericin from XS7, XS8 and Qiubai

**Figure 4:**
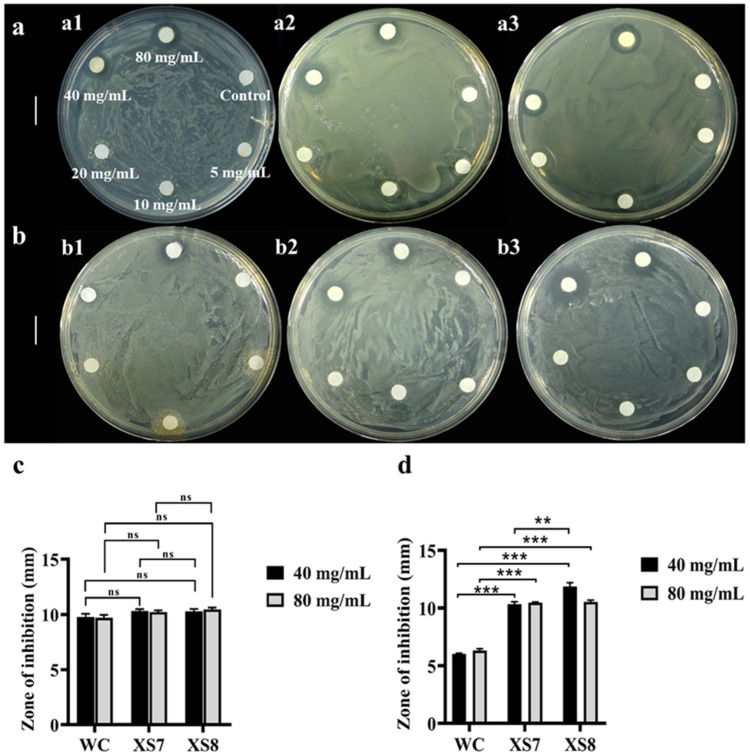
Antibacterial effects of sericin. (**a1**–**a3**) White cocoon sericin, XS7 sericin, and XS8 sericin against *E. coli*, respectively; (**b1**–**b3**) white cocoon (Qiubai) sericin, XS7 sericin and XS8 sericin against *S. aureus*, respectively; (**c**) antibacterial zone size of *E. coli*; (**d**) antibacterial zone size of *S. aureus*. The layout of filter paper discs on each plate was similar to (**a1**). Each bar represents the mean ± SD of three samples. **, *p* < 0.01; ***, *p* < 0.001; ns, no significance. Scale bar: 1 cm.

Given the outstanding antimicrobial activity demonstrated by white sericin [27] and carotenoids [12], it is enticing to investigate whether XS7 and XS8 sericin exhibit stronger antimicrobial activity than Qiubai sericin. Through antibacterial experiments, Qiubai sericin manifested an inhibitory effect on *E. coli* at the concentration of 80 mg/mL or 40 mg/mL. We could not observe significant inhibition circles when the concentration of Qiubai sericin was equal to 20 mg/mL or less (Figure 4a). On the other hand, Qiubai sericin almost showed no inhibition effect on *S. aureus* when the concentration was up to 40 mg/mL and 80 mg/mL (Figure 4b). XS7 and XS8 sericin showed similar inhibitory effects on *E. coli* (Figure 4a) and had a better inhibition effect on *S. aureus* at a concentration of 80 mg/mL and 40 mg/mL (Figure 4b). These results indicated that sericin extracted from XS7, XS8, and Qiubai all showed a good antibacterial effect on *E. coli* (Figure 4c), whereas XS7 and XS8 sericin exhibited a better inhibitory effect on *S. aureus* than Qiubai sericin (*p* < 0.001) (Figure 4d). A remarkable observation was that 40 mg/mL XS8 sericin displayed a better antibacterial effect on *S. aureus* than XS7 sericin at the same concentration (*p* < 0.01) (Figure 4b,d).

### 2.5. Anticancer Capability and Biocompatibility of Sericin from XS7, XS8, and Qiubai

**Figure 5:**
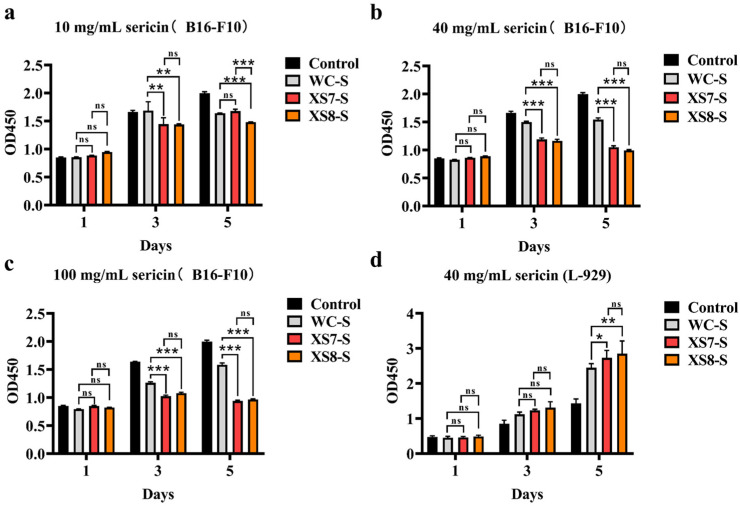
Biocompatibility testing of sericin: (**a**) 10 mg/mL sericin (B16-F10); (**b**) 40 mg/mL sericin (B16-F10); (**c**) 100 mg/mL sericin (B16-F10); and (**d**) 40 mg/mL sericin (L-929). Control, blank without sericin; WC-S, white cocoon (Qiubai) sericin; XS7-S, XS7 sericin; XS8-S, XS8 sericin. Each bar represents the mean ± SD of three samples. *, *p* < 0.05; **, *p* < 0.01; ***, *p* < 0.001; ns, no significance.

We investigated whether sericin showed an inhibitory effect on cancer cell growth (Figure 5). On the 1st day, B16-F10 cell activity showed no significant differences among all groups for all concentrations. On the 3rd day, XS7, XS8, and Qiubai sericin all showed significant inhibitory effects on B16-F10 cell activity compared with the control group. On the 5th day, all experiment groups showed significant inhibitory effects on B16-F10 cell activity compared with the control group. Note that XS7 and XS8 sericin displayed better inhibitory effects on the activity of B16-F10 cells than Qiubai sericin (Figure 5). Interestingly, XS8 sericin of 10 mg/mL had a more significant inhibitory effect compared with XS7 and Qiubai sericin of 10 mg/mL on the 5th day (*p* < 0.001) (Figure 5a). We also assessed whether sericin inhibited normal somatic cell growth with CCK-8 cytotoxicity assay using the L-929 cells (Figure 5d). On the 1st day, cell viability displayed no differences among all groups. On the 3rd day and 5th day, sericin significantly promoted the L-929 cell proliferation. These results suggested that sericin possessed favorable biocompatibility, and XS7 and XS8 sericin had better cell compatibility than Qiubai sericin (Figure 5).

### 2.6. Comparison of Antioxidant Properties of Sericin from XS7, XS8, and Qiubai

**Figure 6:**
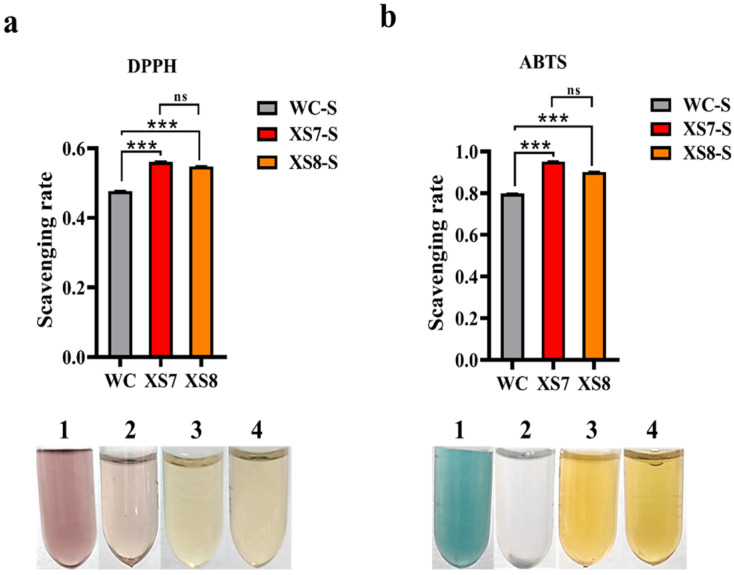
Antioxidant properties testing of sericin. (**a**) DPPH; (**b**) ABTS. WC-S, white cocoon sericin; XS7-S, XS7 sericin; XS8-S, XS8 sericin. 1, Blank control; 2, White cocoon (Qiubai) sericin; 3, XS7 sericin; 4, XS8 sericin. Each bar represents the mean ± SD of three samples. ***, *p* < 0.001; ns, no significance.

Both sericin and carotenoids are recognized for their excellent antioxidant properties [28]. The antioxidant properties of natural carotenoid-containing sericin derived from yellow–red cocoons were evaluated (Figure 6). Qiubai sericin exhibited a good scavenging capacity for DPPH with a scavenging rate of 47.6%, whereas XS7 and XS8 sericin showed significantly higher scavenging ability for DPPH with a scavenging rate of 56.0% and 54.9%, respectively (Figure 6a). The scavenging rate of XS7 and XS8 sericin for ABTS was also significantly higher than Qiubai sericin (Figure 6b).

## 3. Discussion

This is the first report looking into the application potential of natural carotenoid-containing sericin derived from yellow–red cocoons of *B. mori* as a biomass material. Previous studies have demonstrated many excellent properties of sericin derived from commercial white cocoons, including antioxidant properties, antibacterial properties, UV protection, good water solubility, and moisture retention [29], as a biomaterial. A lot of research has also confirmed the potential health benefits of carotenoids for humans, such as anticancer, anti-inflammatory, antibacterial, antidiabetic, and neuroprotective [12]. For *B. mori*, in addition to white cocoons, there are also yellow–red cocoons produced by rare local strains. These yellow–red cocoons are rich in natural carotenoids [14,15], thus offering a new kind of natural carotenoid-containing sericin. Our work demonstrated that natural carotenoid-containing sericin had a better antibacterial effect, anti-cancer ability, cytocompatibility, and antioxidant activity than white cocoon sericin, suggesting a better application potential of natural carotenoid-containing sericin than frequently used white cocoon sericin.

Our work uncovered some special physicochemical characteristics in natural carotenoid-containing sericin compared with white cocoon sericin, as well as the mixture of white cocoon sericin with lutein and β-carotene, which may lead to the better antibacterial effect, anti-cancer ability, cytocompatibility, and antioxidant activity. SEM observation revealed more pores in natural carotenoid-containing sericin than in white cocoon sericin, which may result from the phase separation of water and sericin due to the addition of hydrophobicity carotenoids. The decrease in peak intensity at 1536 cm^−1^ (C-H stretching vibration and N-H bending vibration), 1400 cm^−1^ (C-N stretching vibration), and 1238 cm^−1^ (C-N stretching vibration) in natural carotenoid-containing sericin suggested that the amide II and amide III bands are reduced. The enhancement at peaks of 1640 cm^−1^ (C=C stretching vibration) and 1616 cm^−1^ (C=O stretching vibration) meant that the amide Ι band is increased in natural carotenoid-containing sericin. We further analyzed the protein secondary structure of sericin materials using the software Peakfit. We found that compared to white cocoon sericin, XS7 and XS8 sericin had a decrease in random curling content and a significant increase in β-sheet content. There are a large number of hydrogen bonds in β-sheet to promote structure stability. The addition of natural carotenoids promoted the transition from random coil to β-sheet, which was conducive to improving the gel properties of sericin. This is a new discovery in the research of sericin. We speculated that due to the natural addition of carotenoids, the molecular structure of sericin rearranged and formed more hydrogen bonds. The change in chemical structure further generated the imparity of luminescent property. A special emission band near 564 nm was found in natural carotenoid-containing sericin, but not in white cocoon sericin as well as the mixture of white cocoon sericin with lutein and β-carotene, further demonstrating the interaction of carotene with sericin at the molecular level. Lutein and β-carotene are isoprene compounds, forming conjugated systems with C=C. As an electron donor, the conjugated system can form hydrogen bonds with the amino and carboxyl groups of sericin [30]. In addition, there are hydroxyl groups in lutein molecules, which can also form hydrogen bonds with sericin molecules [31]. Therefore, we speculate that hydrogen bonding between sericin and carotenoids endows XS7 and XS8 sericin proteins with unique fluorescence properties. The interaction might occur during silkworm spinning [32]. The molecular interaction produced during the silkworm spinning may enhance the dissolving capacity of carotenoids and endow a symmetrical distribution of carotenoids in sericin. Many composition materials have been developed to obtain better properties by means of synergistic effects among materials [33,34]. The more excellent properties of natural carotenoid-containing sericin obviously resulted from the synergistic effect of sericin with carotenoids, each of which also had commendable biocompatibility, inoxidizability, and antibacterial properties [12]. These results suggested that natural carotenoid-containing sericin derived from strains XS7 and XS8 is a natural hybrid material between sericin and carotenoids, rather than a simple mixture of sericin and carotenoids, and thus displays more excellent characteristics than white cocoon sericin and carotenoids. The natural hybrid material between sericin and carotenoids may be a better candidate as a scaffold material, antibacterial agent, anticancer carrier, and antioxidant than white cocoon sericin.

This study also found some differences in fluorescence characteristics, antimicrobial effects, anti-cancer ability, and antioxidants between XS7 and XS8 sericin. Previous studies have speculated that Cameo2 and SCRB15 contained the structural domains of specific recognition of lutein and β-carotene, which leads to the selective binding of Cameo2 and SCRB15 to lutein and β-carotene, respectively [17]. The results of qRT-PCR and HPLC in this study confirmed that XS7 and XS8 exhibited significant differences in the expression of the two genes related to carotenoid absorption, which indicated that after the selection of 12 generations, XS7 and XS8 have become two yellow–red cocoon silkworm strains with significant differences. The CD spectra detection revealed significant differences in the positions and intensities of the characteristic peak between XS7 and XS8 sericin, and the fluorescence spectra detection found certain structural differences in the protein structures of XS7 and XS8 sericin, further highlighting the variability between XS7 and XS8 strains. The application results also demonstrated that XS8 sericin exhibited better inhibitory effects on S. aureus and B16-F10 cells than XS7 sericin, whereas XS7 sericin showed better DPPH and ABTS radical scavenging effects than XS8 sericin, which might be attributed to the differences in the carotenoid composition and content between XS7 and XS8 sericin. Collectively, these results provide insights into future breeding efforts in the carotenoid-specific silkworm strains.

## 4. Materials and Methods

### 4.1. Silkworms

Two yellow–red cocoon strains, named XS7 and XS8, derived from one common ancestor for over 12 generations were used in this study. The two strains only had four instar and they were maintained in our laboratory at the Southwest University of China. A white cocoon strain Qiubai was also used as the control. The larvae were nurtured on fresh mulberry leaves at a consistent temperature of 25 °C under a natural light/dark cycle. Their cocoons, silk glands, and related tissues were collected and stored at −80 °C for further use.

### 4.2. Morphological and Molecular Comparison of the Silk Glands of XS7 and XS8 Silkworms

Silk glands were procured from larvae at various stages by the dissecting technique and observed under a microscope (SZX10, OLYMPUS, Tokyo, Japan). The quantitative reverse transcription polymerase chain reaction (qRT-PCR) method was also used to examine the expression dynamics of *Cameo2* and *SCRB15* in the silk glands during the developmental stages of XS7 and XS8. Cameo2 and SCRB15, two members of the scavenger receptor family, allow specific selective transmission of lutein or β-carotene to the middle silk glands of the silkworm, respectively [15]. Briefly, Trizol reagent (Takara, Shiga, Japan) was utilized for RNA extraction, then RT-PCR was conducted based on the RNA template using M-MLV reverse transcriptase (Promega, Madison, WI, USA). The subsequent quantitative PCR (qPCR) was performed for 40 cycles utilizing a Mix (Vazyme, Nanjing, China) as outlined in the instructions. Primers for qRT-PCR were generated using Primer Premier 5.0 Software (Premier, Vancouver, BC, Canada) (Table 2). The relative gene expression levels were calculated using the 2^−ΔCt^ with *rpl3* serving as the internal reference gene [35].

### 4.3. Carotenoid Extraction and Determination

Silk glands were intertwined with carotenoid extract solution (*w*:*v* = 1:10; extract solution: N-hexane:ethanol:acetone = 2:1:1) (CDCHEM, Chongqing, China) and sonicated for 20 min at 4 °C (1.5 s on, 3 s off, 40 W) [18,19]. Subsequently, the concoction was centrifuged at 6500× *g* for 10 min at 4 °C until the solution stratification was achieved. The upper phase was transferred to a new centrifuge tube, and then an equal volume of extract solution was added to the lower phase. This operation was repeated until the upper phase was colorless (more than 3 times). The upper phase solution was vacuum-dried for 1 h. The drying product was blended with MTBE (methyl tert-butyl ether) (Fisher Chemical, Waltham, MA, USA) and saponification solution at a volume ratio of 1:1 and saponified in the dark for more than 10 h.

Cocoon fragments were incorporated with 0.5% sodium carbonate solution (*w*:*v* = 1:40) (Aladdin, Shanghai, China) and heated in a water bath at 90 °C for 1 h. The carotenoid extracting solution (N-hexane:ethanol:acetone = 2:1:1) (CDCHEM, Chongqing, China) was added into the solution above (*v*:*v* = 1:1) followed by subjecting the mixture to sonication for 20 min at 4 °C (1.5 s on, 3 s off, 40 W) [36,37].

The resulting solution was then centrifuged at 6500× *g* for 10 min at 4 °C until the solution was stratified. The upper phase solution was collected and vacuum-dried to obtain carotenoid powder. The carotenoid powder was dissolved with MTBE and filtered with 0.22 μm filter membranes.

A high-performance liquid chromatographic instrument (HPLC, E2695, Waters, Milford, MA, USA) was utilized to identify the content and type of carotenoid. A C30 carotenoid special analytical column (YMC, Kyoto, Japan) was used to separate samples. Methanol, acetonitrile, and MTBE of HPLC grade were used as the mobile phase. The flow rate was 1 mL/min. The samples were detected in triplicates at wavelength 450 nm.

### 4.4. Extraction of Sericin from Cocoons

Cocoons were submerged in 0.5% sodium bicarbonate solution (*w*:*v* = 1:50) (Aladdin, Shanghai, China) and heated at 90 °C for 30 min [16]. The process was repeated once more. Solutions resulting from the above twice-degumming process were mixed, and the suspending impurities were removed firstly by filter paper, followed by filtration through 0.45 μm membranes (Millipore, Boston, MA, USA). Further, the degumming solution was dialyzed with deionized water in an 8000–12,000 Da dialysis membrane (ACMEC, Shanghai, China) for 3 days, during which, the deionized water was substituted every 6 h. The dialyzed degumming solution was then condensed using a rotary evaporator (RE-52AA, LOIKAW, Yancheng, China) at 60 °C. The final sericin solution was frozen at −80 °C for 4 h and then freeze-dried for 48 h to obtain dry powder.

### 4.5. Characterization of Sericin

A Phenom Pro (Phenom World, Eindhoven, The Netherlands) scanning electron microscopy (SEM) instrument was used to observe the external morphology of sericin. The samples were sprayed with gold for 90 s. The surface morphology of sericin was observed under the conditions of 10 kV acceleration voltage.

Sericin was mixed with KBr (*w*:*w* = 1:100) and detected by a Fourier transform infrared (FT-IR) spectrum analyzer (Nicolet iS, Thermo Fisher, Waltham, MA, USA). The scanning wavelength range was 4000–500 cm^−1^. Samples were scanned 300 times at room temperature with a scanning interval of 1 cm^−1^. The protein secondary structure of samples was analyzed by using Systat PeakFit v4.12 (Cranes Software, San Jose, CA, USA).

A Raman spectrometer (DXR2, Thermo Fisher, Waltham, MA, USA) was used to detect the chemical structure of sericin. Each sample was scanned for 100 times in the scanning range of 50–3000 cm^−1^.

The circular dichroic (CD) spectral characteristic of sericin (concentration of 0.5 mg/mL) was evaluated at 190–250 nm and 25 °C by a J-810 CD spectrometer (Jasco, Hachiōji, Japan).

Each sample was ground into a powder and filled into the glass sample rack. The tablet was pressed evenly. Diffraction angles of 0–50° were examined by an X-ray diffraction (XRD) instrument (TD-3500, Dandong Tongda Science & Technology Co., Ltd., Dandong, China).

The excitation wavelength was set at 340 nm, and the emission wavelength was detected by the fluorescence spectrometer (Aqualog, Horiba, Tokyo, Japan) spanning 350–600 nm with 5 nm intervals.

### 4.6. Antimicrobial Effect Investigation

Firstly, the antimicrobial filter paper discs containing sericin were prepared by adding 20 μL of each sericin sample onto the filter paper discs and drying them in air. Then bacteria were refreshed in the incubator for 12 h (*E. coli*: 37 °C; *S. aureus*: 28 °C). The single colony of bacteria in the medium was inoculated in LB liquid culture medium, and bacteria were cultured overnight in a shaking incubator (200 rpm) at appropriate temperature conditions. After culturing, 150 μL of bacterial solution was evenly spread on the surface of plate containing LB solid medium. The filter paper disc containing sericin was covered on the surface of the bacterial liquid. After standing for 30 min, they were put into the incubator upside down for 24 h, and then the diameter of the antibacterial circle was measured after removing the filter paper. The diameter of the filter paper was 5 mm, and the diameter of the antibacterial circle was measured after removing the filter paper. Therefore, the diameter of antibacterial circle without antibacterial effect was 5 mm.

### 4.7. Anti-Tumor Activity and Cytocompatibility of Sericin

Mouse melanoma B16-F10 cells and mouse fibroblast L-929 cells were used to detect the anti-tumor activity and cytocompatibility, respectively. The mouse melanoma B16-F10 cells were provided by the State Key Laboratory of Ultrasonic Medical Engineering of Chongqing Medical University, and the mouse fibroblast L-929 cells were purchased from the Procell Life Science and Technology Corporation (Wuhan, China). B16-F10 cells were cultured in DMEM culture medium (Gibco, Grand Island, NY, USA) containing 10% FBS (Sangon Biotech, Shanghai, China) and 1% p/s (5% CO_2_, 37 °C). L-929 cells were cultured in the NCTC clone 929 specific culture medium (Procell, Germany) containing 10% FBS and 1% p/s (AIDISHENG, Yangzhou, China) (5% CO_2_, 37 °C).

A CCK-8 kit (Beijing Zoman Biotechnology Co., Ltd., Beijing, China) was employed to detect the anti-tumor activity of sericin on the proliferation of mouse melanoma B16-F10 cells and the cytocompatibility of the sericin on the proliferation of mouse fibroblast L-929 cell. Sericin derived from XS7, XS8 (yellow–red cocoon), and Qiubai (white cocoon) of strains were dissolved into water to prepare sericin solutions at a concentration of 10 mg/mL, 40 mg/mL, and 100 mg/mL, respectively. The cell density was 10,000 cells per well for B16-F10 cells and 3000 cells per well for L-929 cells in the cell culture plate.

### 4.8. Antioxidant Effects of Sericin

According to the total antioxidant capacity (T-AOC) Assay Kit (DPPH and ABTS) (Sangon Biotech, Shanghai, China), each sericin sample (2 mg) was dissolved in 500 μL of extraction solution (Sangon Biotech, Shanghai, China) to obtain sericin solution at a concentration of 4 mg/mL. The 500 μL sericin solution was mixed with 500 μL T-AOC reagent I in dark for 20 min, then the reaction solution was centrifuged at 4 °C and 8000× *g* for 5 min. The absorbance at 515 nm for each DPPH sample supernatant and the absorbance at 714 nm for each ABTS sample supernatant were measured immediately using an ultraviolet spectrophotometer (F-4600, HITACHI, Tokyo, Japan). The calculation for the scavenging ratio of DPPH radicals was as follows [38]:

Scavenging activity (%) = (Control_OD_−Sample_OD_)/Control_OD_ × 100%

### 4.9. Statistics Analysis

The significance of difference in data between two groups was analyzed by the Turkey Test of Origin 2021 (OriginLab, Northampton, MA, USA). The significance of differences within groups was evaluated by one-way analysis of variance (NOVA, OriginLab, Northampton, MA, USA).

## 5. Conclusions

This research verifies that XS7 and XS8 are two yellow–red cocoon silkworm strains with differences in cocoon color and the expression of genes related to carotenoid absorption. The addition of carotenoids alters the protein secondary structure of sericin. Compared to white cocoon sericin, the β-sheet increases significantly in XS7 and XS8 sericin. Moreover, the interaction of carotenes with sericin contributes to a special emission at 530 and 564 nm. The addition of carotenoids endows more excellent antibacterial (for *S. aureus*), anti-cancer, cytocompatibility, and antioxidant properties for XS7 and XS8 sericin, which implies they may be better candidates for application in biomedicine, biosensors, food, and cosmetics.

## Figures and Tables

**Table 1:** Secondary structure analysis of sericin samples.

Name	β-Sheet (%)	Random Coil (%)	α-Helix (%)	β-Turn (%)
White cocoon sericin	28.16	16.80	12.12	42.92
XS7 sericin	63.87	11.40	8.74	15.99
XS8 sericin	58.58	14.54	10.47	14.78

**Table 2:** Primers used for qRT-PCR.

Name	Sequence	Length (bp)
*BmSCRB15*-F	TGGAATACCACGGCAATAAGAC	22
*BmSCRB15*-R	ATGGGCAAACCATAAAAGCAAG	22
*BmCameo2*-F	ACAAGCACTCGTTCTATTTCGC	22
*BmCameo2*-R	TCATACAATGTGATGTGGTCGC	22
*Bmrpl3*-F	TTCGTACTGGCTCTTCTCGT	20
*Bmrpl3*-R	CAAAGTTGATAGCAATTCCCT	21

## Data Availability

Data present in this manuscript will be made available on request.

## References

[1] Ghosh S., Pati F. (2023). Decellularized extracellular matrix and silk fibroin-based hybrid biomaterials: A comprehensive review on fabrication techniques and tissue-specific applications. Int. J. Biol. Macromol..

[2] Xing T., He A., Huang Z., Luo Y., Zhang Y., Wang M., Shi Z., Ke G., Bai J., Zhao S. (2023). Silk-based flexible electronics and smart wearable Textiles: Progress and beyond. Chem. Eng. J..

[3] Asakura T., Williamson M.P. (2023). A review on the structure of *Bombyx mori* silk fibroin fiber studied using solid-state NMR: An antipolar lamella with an 8-residue repeat. Int. J. Biol. Macromol..

[4] Saad M., El-Samad L.M., Gomaa R.A., Augustyniak M., Hassan M.A. (2023). A comprehensive review of recent advances in silk sericin: Extraction approaches, structure, biochemical characterization, and biomedical applications. Int. J. Biol. Macromol..

[5] Yang C., Yao L., Zhang L. (2023). Silk sericin-based biomaterials shine in food and pharmaceutical industries. Smart Mater. Med..

[6] Hu D., Li T., Liang W., Wang Y., Feng M., Sun J. (2023). Silk sericin as building blocks of bioactive materials for advanced therapeutics. J. Control. Release.

[7] Liu J., Shi L., Deng Y., Zou M., Cai B., Song Y., Wang Z., Wang L. (2022). Silk sericin-based materials for biomedical applications. Biomaterials.

[8] Kumar Dan A., Aamna B., De S., Pereira-Silva M., Sahu R., Cláudia Paiva-Santos A., Parida S. (2022). Sericin nanoparticles: Future nanocarrier for target-specific delivery of chemotherapeutic drugs. J. Mol. Liq..

[9] Zhang J., Ding Z., Du W., Wang X., Guan Y. (2023). Carotenoids act on coloration and increase immunity and antioxidant activity in the novel “Yongzhang Golden turtle” strain of *Pelodiscus sinensis*. Aquaculture.

[10] Gebregziabher B.S., Gebremeskel H., Debesa B., Ayalneh D., Mitiku T., Wendwessen T., Habtemariam E., Nur S., Getachew T. (2023). Carotenoids: Dietary sources, health functions, biofortification, marketing trend and affecting factors—A review. J. Agric. Food Res..

[11] Razz S.A. (2024). Comprehensive overview of microalgae-derived carotenoids and their applications in diverse industries. Algal Res..

[12] Islam F., Khan J., Zehravi M., Das R., Haque M.A., Banu A., Parwaiz S., Nainu F., Nafady M.H., Shahriar S.M.S. (2024). Synergistic effects of carotenoids: Therapeutic benefits on human health. Process Biochem..

[13] Rehman A., Tong Q., Jafari S.M., Assadpour E., Shehzad Q., Aadil R.M., Iqbal M.W., Rashed M.M.A., Mushtaq B.S., Ashraf W. (2020). Carotenoid-loaded nanocarriers: A comprehensive review. Adv. Colloid Interface Sci..

[14] Ma M., Hussain M., Dong S., Zhou W. (2016). Characterization of the pigment in naturally yellow-colored domestic silk. Dye. Pigment..

[15] Tsuchida K., Sakudoh T. (2015). Recent progress in molecular genetic studies on the carotenoid transport system using cocoon-color mutants of the silkworm. Arch. Biochem. Biophys..

[16] Vaishnav S.R., Singh S.A. (2023). Chapter 14—Sericin, a by-product of the silk industry: Extraction and applications. Value-Addition in Agri-Food Industry Waste Through Enzyme Technology.

[17] Sakudoh T., Kuwazaki S., Iizuka T., Narukawa J., Yamamoto K., Uchino K., Sezutsu H., Banno Y., Tsuchida K. (2013). CD36 homolog divergence is responsible for the selectivity of carotenoid species migration to the silk gland of the silkworm *Bombyx mori*. J. Lipid Res..

[18] Zhu H., Zhang X.-X., Zhang R., Feng J.-Y., Thakur K., Zhang J.-G., Wei Z.-J. (2023). Anti-hardening effect and mechanism of silkworm sericin peptide in high protein nutrition bars during early storage. Food Chem..

[19] Bascou R., Hardouin J., Ben Mlouka M.A., Guénin E., Nesterenko A. (2022). Detailed investigation on new chemical-free methods for silk sericin extraction. Mater. Today Commun..

[20] Zhang L., Hao M., Yao L., Xing C., Wen Q., Zhang Z., Yu J., Wang J., Xing D., Zheng T. (2023). Sericin “hairpin structure”-based multifunctional anthocyanin nanoencapsulation for remodeling ROS-dependent cutaneous wound healing. Chem. Eng. J..

[21] Castrillón Martínez D.C., Zuluaga C.L., Restrepo-Osorio A., Álvarez-López C. (2017). Characterization of sericin obtained from cocoons and silk yarns. Procedia Eng..

[22] Rocha L.K.H., Favaro L.I.L., Rios A.C., Silva E.C., Silva W.F., Stigliani T.P., Guilger M., Lima R., Oliveira J.M., Aranha N. (2017). Sericin from *Bombyx mori* cocoons. Part I: Extraction and physicochemical-biological characterization for biopharmaceutical applications. Process Biochem..

[23] Portarena S., Anselmi C., Leonardi L., Proietti S., Bizzarri A.R., Brugnoli E., Baldacchini C. (2023). Lutein/β-carotene ratio in extra virgin olive oil: An easy and rapid quantification method by Raman spectroscopy. Food Chem..

[24] Oliveira V.E.D., Castro H.V., Edwards H.G.M., Oliveira L.F.C.D. (2010). Carotenes and carotenoids in natural biological samples: A Raman spectroscopic analysis. J. Raman Spectrosc..

[25] Guo K., Zhang X., Zhao D., Qin L., Jiang W., Hu W., Liu X., Xia Q., Dong Z., Zhao P. (2022). Identification and characterization of sericin5 reveals non-cocoon silk sericin components with high β-sheet content and adhesive strength. Acta Biomater..

[26] Bhagath Singh G.V.P., Subramaniam K.V.L. (2016). Quantitative XRD study of amorphous phase in alkali activated low calcium siliceous fly ash. Constr. Build. Mater..

[27] Shitole M., Dugam S., Tade R., Nangare S. (2020). Pharmaceutical applications of silk sericin. Ann. Pharm. Fr..

[28] Zhang R., Yang W., Pan Q., Zeng Q., Yan C., Bai X., Liu Y., Zhang L., Li B. (2023). Effects of long-term blue light irradiation on carotenoid biosynthesis and antioxidant activities in Chinese cabbage (*Brassica rapa* L. ssp. pekinensis). Food Res. Int..

[29] Khamhaengpol A., Siri S. (2017). Composite Electrospun Scaffold Derived from Recombinant Fibroin of Weaver Ant (*Oecophylla smaragdina*) as Cell-Substratum. Appl. Biochem. Biotechnol..

[30] Von Lintig J., Moon J., Babino D. (2021). Molecular components affecting ocular carotenoid and retinoid homeostasis. Prog. Retin. Eye Res..

[31] Cao S., Zhu R., Wu D., Su H., Liu Z., Chen Z. (2024). How hydrogen bonding and π–π interactions synergistically facilitate mephedrone adsorption by bio-sorbent: An in-depth microscopic scale interpretation. Environ. Pollut..

[32] Song K., Wang Y., Dong W., Li Z., Xia Q., Zhu P., He H. (2023). Decoding silkworm spinning programmed by pH and metal ions. Sci. Bull..

[33] Mumtaz S., Ali S., Pervaiz A., Qureshi M.Z., Kanwal K., Saleem T. (2023). Apoptotic and antiproliferative effects of silk protein sericin conjugated-AgNO_3_ nanoparticles in human breast cancer cells. Saudi J. Biol. Sci..

[34] Shalini T., Pavithraa G., Rakkesh R.A., Balakumar S. (2023). Unravelling the nature-inspired silk sericin—Calcium phosphate hybrid nanocomposites: A promising sustainable biomaterial for hard tissue regeneration applications. Surf. Interfaces.

[35] Scoville A.G., Barnett L.L., Bodbyl-Roels S., Kelly J.K., Hileman L.C. (2011). Differential regulation of a MYB transcription factor is correlated with transgenerational epigenetic inheritance of trichome density in *Mimulus guttatus*. New Phytol..

[36] Menezes Silva J.V., Silva Santos A., Araujo Pereira G., Campos Chisté R. (2023). Ultrasound-assisted extraction using ethanol efficiently extracted carotenoids from peels of peach palm fruits (*Bactris gasipaes* Kunth) without altering qualitative carotenoid profile. Heliyon.

[37] Suo A., Fan G., Wu C., Li T., Cong K. (2023). Green extraction of carotenoids from apricot flesh by ultrasound assisted corn oil extraction: Optimization, identification, and application. Food Chem..

[38] Nguyen D.D., Luo L.J., Yang C.J., Lai J.Y. (2022). Highly Retina-Permeating and Long-Acting Resveratrol/Metformin Nanotherapeutics for Enhanced Treatment of Macular Degeneration. ACS Nano.

